# Measuring glucocorticoid receptor expression *in vivo* with PET

**DOI:** 10.18632/oncotarget.24911

**Published:** 2018-04-17

**Authors:** Charles Truillet, Matthew F.L. Parker, Loc T. Huynh, Junnian Wei, Khaled M. Jami, Yung-Hua Wang, Yuqin S. Shen, Renuka Sriram, David M. Wilson, John Kurhanewicz, Michael J. Evans

**Affiliations:** ^1^ Department of Radiology and Biomedical Imaging, University of California San Francisco, San Francisco, CA 94143, USA; ^2^ Helen Diller Family Comprehensive Cancer Center, University of California San Francisco, San Francisco, CA 94143, USA; ^3^ Department of Pharmaceutical Chemistry, University of California San Francisco, San Francisco, CA 94143, USA

**Keywords:** cancer, glucocorticoid receptor, precision medicine, positron emission tomography, pharmacodynamics

## Abstract

The glucocorticoid receptor (GR) is an emerging drug target for several common and deadly solid tumors like breast and prostate cancer, and clinical trials studying the antitumor effects of GR antagonists are beginning. Since GR expression can be variable in tumor cells, and virtually all normal mammalian tissues express some GR, we hypothesized that an imaging tool capable of detecting GR positive tumors and/or measuring GR occupancy by drug in tumor and normal tissues could improve the precision application of anti-GR therapies in the clinic. To this end, we developed a fluorine-18 labeled corticosteroid termed GR02 that potently binds the endogenous ligand binding pocket on full length GR. Binding of ^18^F-GR02 was suppressed in many normal tissues by co-treatment with mifepristone, a GR antagonist in human use, and was elevated in many normal tissues among mice lacking circulating corticosteroids due to adrenalectomy. ^18^F-GR02 also accumulated in GR positive subcutaneous and subrenal capsule prostate cancer models, and uptake in tumors was competed by mifepristone. Combined with a straightforward and high yielding radiosynthesis, these data establish the foundation for near-term clinical translation of ^18^F-GR02.

## INTRODUCTION

The glucocorticoid receptor (GR) is a nuclear hormone receptor that regulates many cellular processes, including catabolism and apoptosis [1, 2]. The transcriptional activity of GR in peripheral tissues is activated by binding to corticosteroids, which are synthesized and secreted by the adrenal cortex. Corticosteroid production is controlled by a well understood negative feedback endocrine loop termed the hypothalamus-pituitary-adrenocortical axis. In healthy organisms, a pulse of high corticosteroid production and secretion typically occurs transiently after periods of stress, whereupon homeostasis is restored by corticosteroid metabolism in peripheral tissues [2].

While changes in GR expression levels and activity have long been suspected to contribute to the pathobiology of human disorders like anxiety and depression, more recently it has become clearer that GR may have a role in cancer morbidity. For instance, hyperactivation of GR in tumor cells overrides the effects of cytotoxic chemotherapy in breast and ovarian cancer [3–6]. Moreover, high expression of GR in newly diagnosed triple negative breast cancer appears to result in an especially fatal form of this already aggressive subtype [7]. Although early stage prostate cancer does not express GR, upregulation of GR in castration resistant prostate cancer was recently identified as mechanism of resistance to the antiandrogen enzalutamide (Xtandi) [8–10], as GR can transcriptionally activate direct androgen receptor (AR) target genes, but enzalutamide cannot bind and inactivate GR. In concert with GR expression, deficient corticosteroid metabolism in castration resistant prostate cancer cells can also override the antitumor activity of enzalutamide [10].

Encouragingly, several groups have shown that GR antagonists or inhibitors of BET bromodomain containing protein 4 can re-sensitize many of the abovementioned cancers to standard of care systemic therapies [11–15]. On this basis, several clinical trials have been recently opened to test the clinical benefit of GR antagonists (e.g. mifepristone/RU486, CORT125134) as single agents or in combination with standard of care therapy for several late stage cancers (www.clinicaltrials.gov).

Especially considering GR expression in tumors has been shown to be quite variable [7, 8], identifying treatment naïve patients whose tumors express GR will likely be crucial to definitively assessing the clinical benefit of GR inhibition. Moreover, unlike other “druggable” nuclear hormone receptors within the same subfamily (e.g. AR, estrogen receptor [ER]), GR is not selectively expressed or perhaps even overexpressed in cancer compared to normal tissues. Therefore, understanding the on-target, off-tissue pharmacology of experimental GR antagonists will likely be essential for predicting leads with optimal clinical activity and minimal toxicity.

While there is currently no gold standard non-invasive diagnostic assay for identifying GR-expressing tumors, the tumor-autonomous expression of other nuclear hormone receptors within the same subfamily (e.g. AR, ER, progesterone receptor [PR]) has been successfully detected in patients with radiolabeled agonists and positron emission tomography (PET) [16–18]. On this basis, we sought to develop a radioligand that could detect GR expression levels in tumors.

## RESULTS

**Figure 1:**
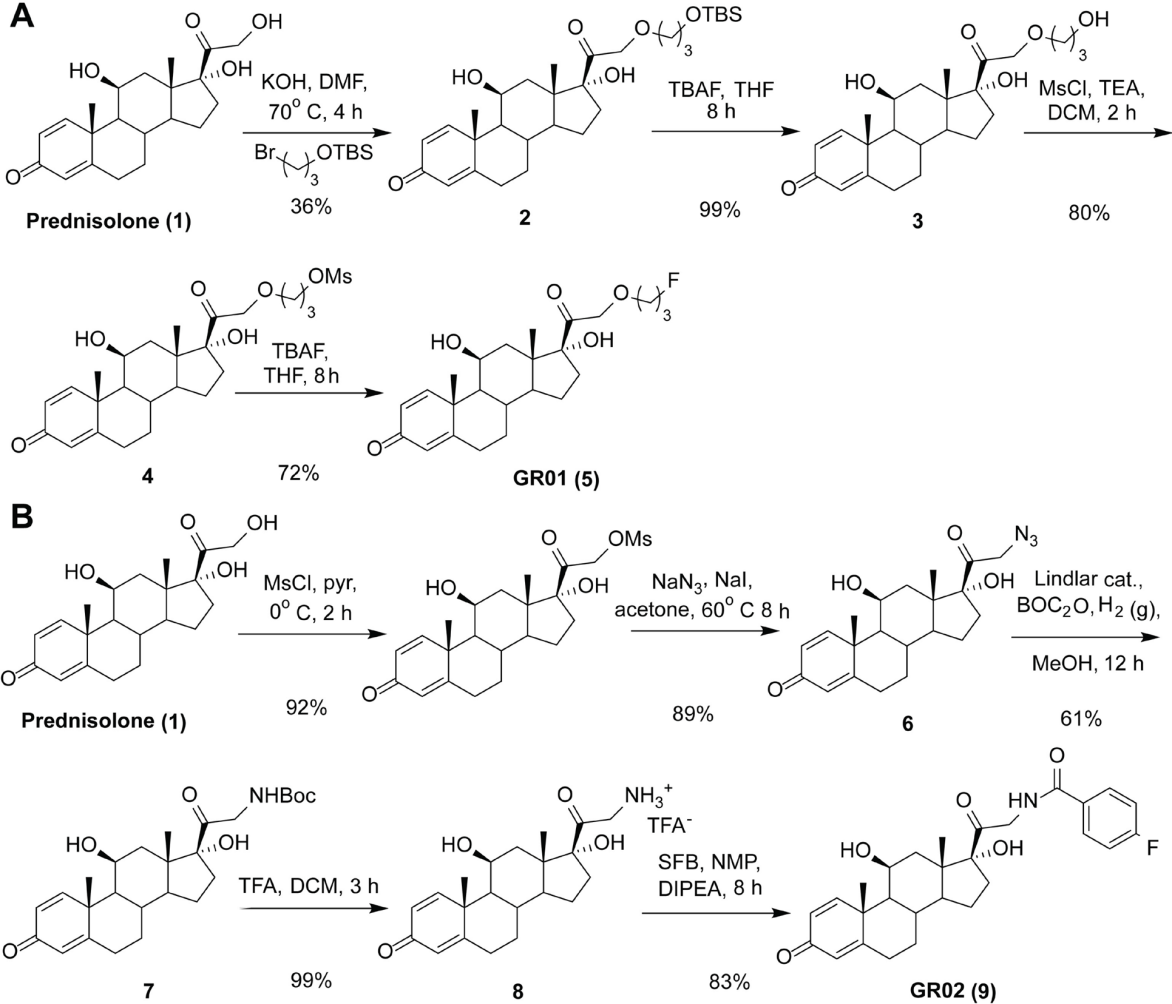
Synthesis of fluorinated ligands for GR (**A**) A scheme outlining how GR01 was synthesized from prednisolone. 3-bromopropyl-*tert*-butyldimethylsilyl ether was appended to the C21 alcohol on prednisolone, and after silyl deprotection, the primary alcohol was activated for nucleophilic displacement with methanesulfonyl chloride. The resulting mesylate was displaced with a nucleophilic source of fluorine-19 to confer GR01. (**B**) A scheme showing how GR02 was synthesized from prednisolone. The C21 primary alcohol was activated for nucleophilic attack as a mesylate, converted to 21-aza-prednisolone via displacement with N_3_ anion, and subsequently reduced to a primary amine. The primary amine was coupled to ^19^F-SFB to confer GR02. Full synthetic details and characterization for ^18/19^F-GR01 and ^18/19^F-GR02 are available in the Supplementary Materials.

Prior medicinal chemistry suggested that unnatural chemical functionalities can be installed off of the C17 position of corticosteroids without pejoratively impacting specificity and potency for GR [19–21]. However, alkyl fluorides that bore a fluorine-18 atom adjacent to the C17 position were metabolically unstable *in vivo*, and subject to radiodefluorination. We hypothesized that installing a fluorine atom on a less reactive carbon center branching off of the C17 position would curb radiodefluorination. To this end, we designed and synthesized GR01, which bears an alkyl fluoride on the distal C24 carbon (Figure 1A). 3-Bromo-1-propanol was protected as a tert-butyl silyl ether, and coupled to the C21 primary alcohol on prednisolone (1) via S_N_2 displacement of the alkyl bromide. The silyl ether group on 2 was removed with tetra-butyl ammonium fluoride (TBAF) to confer 3, and the C24 primary alcohol was activated by reaction with methane sulfonyl chloride (MsCl) to confer 4. Nucleophilic displacement of the mesylate leaving group with fluoride anion resulted in ^19^F-GR01 (5) in 20% overall yield (see Supplementary Methods for a full description of synthetic details and characterization).

Aryl fluorides are also well established to be less prone to radiodefluorination *in vivo*. Therefore, we designed and synthesized another GR radioligand, termed GR02 (Figure 1B). The C21 primary alcohol on prednisolone was activated with methane sulfonyl chloride, and converted to 21-aza-prednisolone (6) via nucleophilic displacement with N_3_^-^_._ The azide group was reduced to a primary amine and reacted with di-tert-butyl dicarbonate (BOC_2_O) *in situ* to confer the BOC protected amine 7. Immediately prior to further manipulating 7 synthetically, the BOC group was removed with trifluoroacetic acid (TFA), and the primary amine 8 was isolated as a TFA salt. 8 was reacted with *N*-succinimidyl-4-fluorobenzoate (SFB) to confer ^19^F-GR02 (9) with an overall yield of 41% (see Supplementary Methods for a full description of synthetic details and characterization).

The affinity and selectivity of GR01 and GR02 for subfamily 3 group C nuclear hormone receptors was evaluated with competition binding assays on cells expressing the respective receptor (Table 1). GR01 had a low nanomolar K_D_ for human GR (3.8 nM), and at least 100 fold higher K_D_ for AR, ER, PR, and the mineralocorticoid receptor (MR). GR02 was slightly less potent for GR (K_D_ = 15.9 nM) but selective. Both compounds had a similar affinity for GR as the synthetic agonist dexamethasone (K_D_ = 2.0 nM). GR02 also had a low nM affinity for full length mouse and rat GR.

**Table 1:** A summary of the K_D_ data calculated for GR01 and GR02

Ligand	huGR	huAR	huPR	huER	huMR	msGR	rtGR
Ref.	2.03 × 10^–9^	4.96 × 10^–10^	6.27 × 10^–10^	6.94 × 10^–9^	1.41 × 10^–8^	ND	ND
GR01	3.86 × 10^–9^	1.46 × 10^–6^	1.06 × 10^–6^	9.78 × 10^–7^	2.12 × 10^–7^	ND	ND
GR02	1.59 × 10^–8^	1.26 × 10^–6^	2.42 × 10^–7^	2.07 × 10^–5^	4.03 × 10^–6^	6.71 × 10^–9^	6.18 × 10^–9^

Data were calculated with competition binding assays displacing ^3^H-steroid agonists for the respective human (hu), mouse (ms), and rat (rt) nuclear hormone receptor on cells. Data are reported as [M]. Reference compounds (Ref.) are positive control agonists or antagonists: dexamethasone (GR), dihydrotestosterone (AR), progesterone (PR), 4-OH-tamoxifen (ER), and eplerenone (MR). ND = not determined.

We next conducted radiochemistry and pilot animal imaging studies with GR01 and GR02. The mesylate 4 was reacted with [^18^F]-fluoride anion in anhydrous acetonitrile to confer ^18^F-GR01. ^18^F-GR01 was purified with reverse phase HPLC coupled to a radiation detector, and the decay corrected radiochemical yield was ∼2% (see Supplementary Methods, Supplementary Figure 1, and Supplementary Table 1). ^18^F-SFB was synthesized by hand according to literature precedent [22]. ^18^F-SFB was ligated to 8 in anhydrous acetonitrile to confer ^18^F-GR02. ^18^F-GR02 was isolated by reverse phase HPLC coupled to a radiation detector, and the decay corrected radiochemical yield was ∼20–30% with a specific activity of ∼33–37 GBq/μmol (Supplementary Figure 2 and Supplementary Table 1).

**Figure 2:**
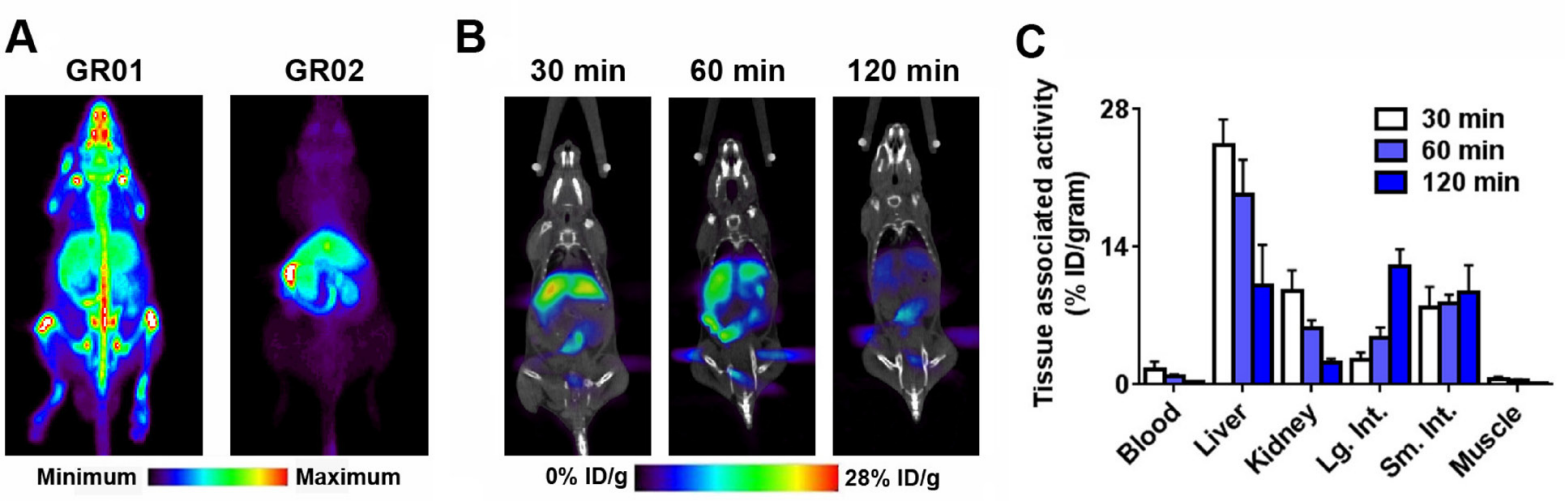
*In vivo* biodistribution of ^18^F-GR01 and ^18^F-GR02 in tumor naïve, intact male C57BL6/J mice (**A**) Maximum intensity projections derived from dynamic acquisitions of ^18^F-GR01 or ^18^F-GR02 show the biodistribution of the radiotracers in representative mice from 50 - 60 minutes post injection. While both radiotracers show accumulation in abdominal tissues known to be GR positive, ^18^F-GR01 is distinguishable from ^18^F-GR02 on the basis of its visually higher bone uptake, which may suggest radiodefluorination *in vivo*. (**B**) Representative coronal PET/CT slices showing the biodistribution of ^18^F-GR02 at three time points post injection in intact male C57BL6/J mice (*n =* 5/time point). (**C**) Biodistribution studies showing the accumulation of ^18^F-GR02 in representative tissues and compartments over time (*n =* 5/time point).

^18^F-GR01 was administered to tumor naïve immunocompetent intact male C57BL6/J mice (∼11 MBq/mouse, *n =* 5) and its biodistribution was evaluated one hour post injection with PET/CT. There was visual evidence of binding to abdominal tissues expected to harbor GR (e.g. liver, kidneys); however, a substantial amount of bone associated activity was observed, suggestive of radiodefluorination (Figure 2A). A biodistribution study 60 minutes post injection also revealed an undesirably high degree of bone associated activity (∼20% ID/g, see Supplementary Figure 3).

In contrast to ^18^F-GR01, PET/CT of tumor naïve intact male C57BL6/J mice (∼11 MBq/mouse, *n =* 5) injected with ^18^F-GR02 did not show visually obvious evidence of activity in the bone at one hour post injection (Figure 2A). Radiotracer accumulation in the liver, kidneys, small and large intestines was visually obvious on PET by eye. A more systematic analysis of radiotracer biodistribution using a one hour dynamic PET scan showed rapid accumulation of ^18^F-GR02 in the liver peaking within 10 minutes post injection, with continuous but slow washout from 10–60 min (Supplementary Figure 4). A typical blood pool time activity curve was observed, and activity in the muscle was low.

These data advocated to us for continued studies with ^18^F-GR02 rather than ^18^F-GR01, and static PET acquisitions were next acquired in a separate cohort of tumor naïve intact male C57BL6/J mice over a broader window of time to identify the optimal time point post injection to study ^18^F-GR02 pharmacology (∼11 MBq/mouse, *n =* 5 mice/time point). Visual inspection of images acquired at 30, 60, and 120 minutes post injection suggested the radiotracer distributed into peripheral tissues from 30 – 60 minutes post injection. The overall intensity of the images diminished substantially from 60 - 120 minutes, suggesting that ^18^F-GR02 was cleared from tissues and/or catabolized during this time (Figure 2B).

A biodistribution study at 30, 60 and 120 minutes post injection showed that the highest uptake of the radiotracer occurred in the liver, kidney, small and large intestine (Figure 2C and Supplementary Figure 5). Low activity was observed in the bone, as expected based on the PET data. Overall, ^18^F-GR02 appeared to accumulate in tissues out to 60 minutes post injection, followed by a reduction in tissue-associated activity from 60 – 120 minutes with a few exceptions (e.g. small and large intestine). These data suggested to us that ^18^F-GR02 would be best studied at 60 minutes post injection.

**Figure 3:**
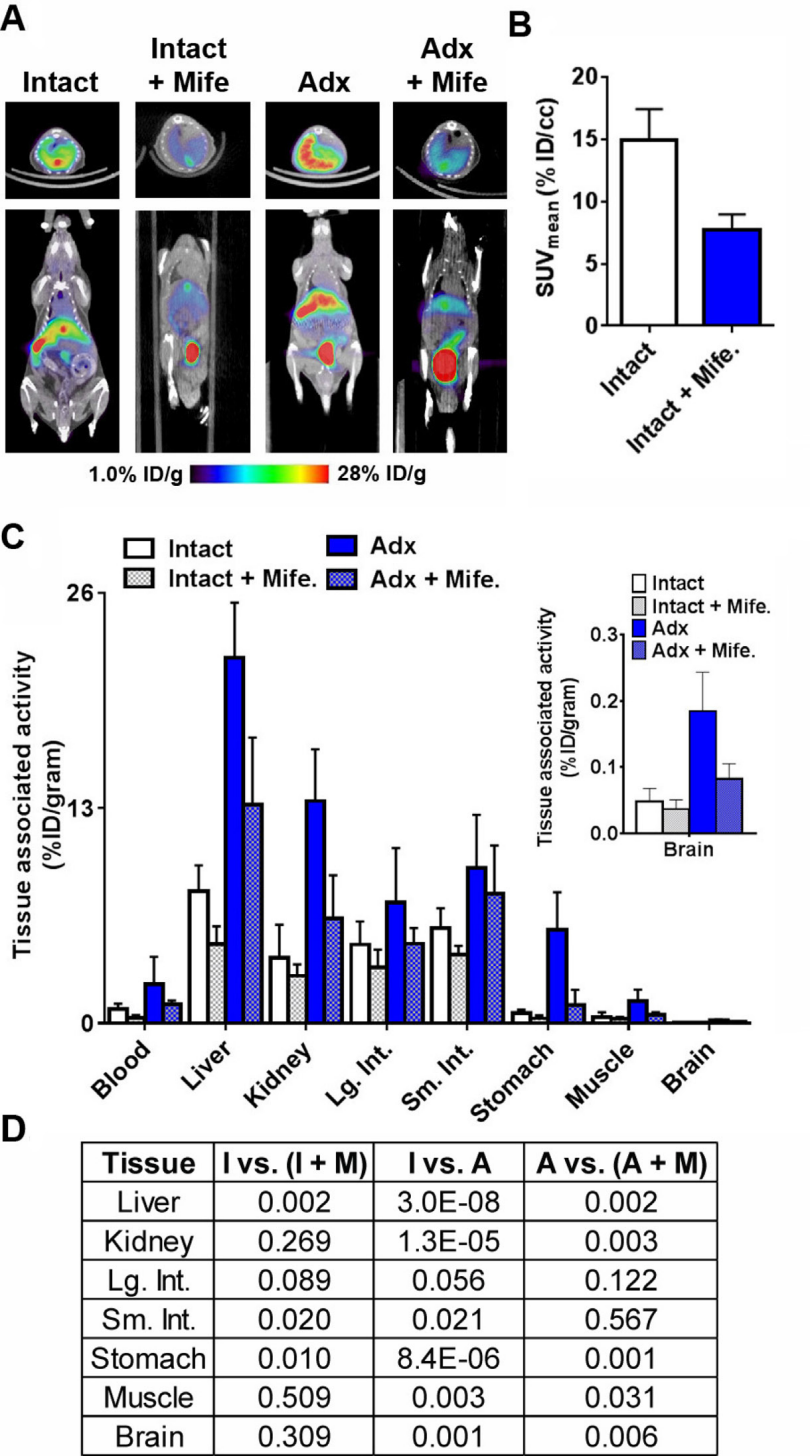
Evidence for the GR specific accumulation of ^18^F-GR02 in tumor naïve C57BL6/J mice (**A**) Representative coronal and transaxial small animal PET/CT images showing ^18^F-GR02 accumulation in the abdominal tissues of tumor naïve intact or adx C57BL6/J mice (*n =* 5/treatment arm). Decay corrected images show that pretreatment of mife. via oral gavage (25 mg/kg) for four days prior to the injection of ^18^F-GR02 suppresses radiotracer uptake in the liver compared to treatment naïve intact or adx mice. Moreover, radiotracer uptake in the liver of adx mice without circulating corticosteroids is higher compared to levels in intact mice, consistent with a model by which ^18^F-GR02 accumulates in tissues due (in part) to direct GR binding in the ligand binding domain. All data were collected 60 minutes post injection of ^18^F-GR02. (**B**) Region of interest analysis shows that the suppression of ^18^F-GR02 uptake in the liver by mife. treatment is sufficiently large enough to be quantifiable using PET data. (**C**) Biodistribution data from select tissues showing the relative changes in ^18^F-GR02 accumulation in intact versus adx mice, and vehicle versus mife. treated mice. The levels in the brain are highlighted in the graphical inset, and the full biodistribution is reported in the Supplementary. (**D**) A grid representing the *P* values calculated for selected tissue to tissue comparisons between treatment arms. Statistically significant changes were interpreted to be *P* < 0.05. Abbreviations: I = intact, A = adrenalectomized, M = mifepristone, Lg. Int. = large intestine, Sm. Int. = small intestine.

We next tested whether ^18^F-GR02 uptake in peripheral tissues was due to specific GR binding. Tumor naïve intact male C57BL6/J mice (*n =* 5/treatment arm) were treated with vehicle or the GR antagonist mifepristone (mife., 25 mg/kg) via daily oral gavage for four days prior to radiotracer injection. One hour post injection of ^18^F-GR02 (∼11 MBq/mouse), its biodistribution was studied with PET/CT and post mortem tissue analysis. The PET/CT data showed that mife. treatment suppressed radiotracer uptake in abdominal tissues like the liver (Figure 3A). The reduction in liver uptake of ^18^F-GR02 was also substantial enough to be quantified with region of interest analysis (Figure 3B and Supplementary Figure 6). A biodistribution study showed statistically significant suppression of ^18^F-GR02 uptake due to mife. treatment in the liver, spleen, small intestine, and stomach (Figure 3C and 3D, see also Supplementary Figure 7).

Because mife. also is a potent ligand for PR, we further probed for evidence of GR-specific binding of ^18^F-GR02 in adrenalectomized (adx) mice lacking circulating corticosteroids (∼11 MBq injected/mouse). Statistically higher radiotracer accumulation was observed via biodistribution studies in several tissues from male adx C57BL6/J mice compared to the respective tissues in intact male mice, as expected (*n =* 5, Figure 3A, 3C and 3D, see also Supplementary Figure 7). Treating adx C57BL6/J mice (*n =* 5) with mife. also suppressed ^18^F-GR02 uptake to a statistically significant extent in several tissues, including liver, kidney and stomach (Figure 3A, 3C and 3D, see Supplementary Figure 7).

Across the treatment arms, ^18^F-GR02 was detectable in the brain of C57BL6/J mice (∼0.05–0.2% ID/g, Figure 3C, inset). Moreover, ^18^F-GR02 levels in the brain of adx was ∼40 fold higher than in the brain of intact mice, and this increase in radiotracer retention was reversible with mife. treatment (Figure 3C and 3D). Collectively, these data suggest that ^18^F-GR02 may traverse the blood brain barrier to specifically bind GR.

**Figure 4:**
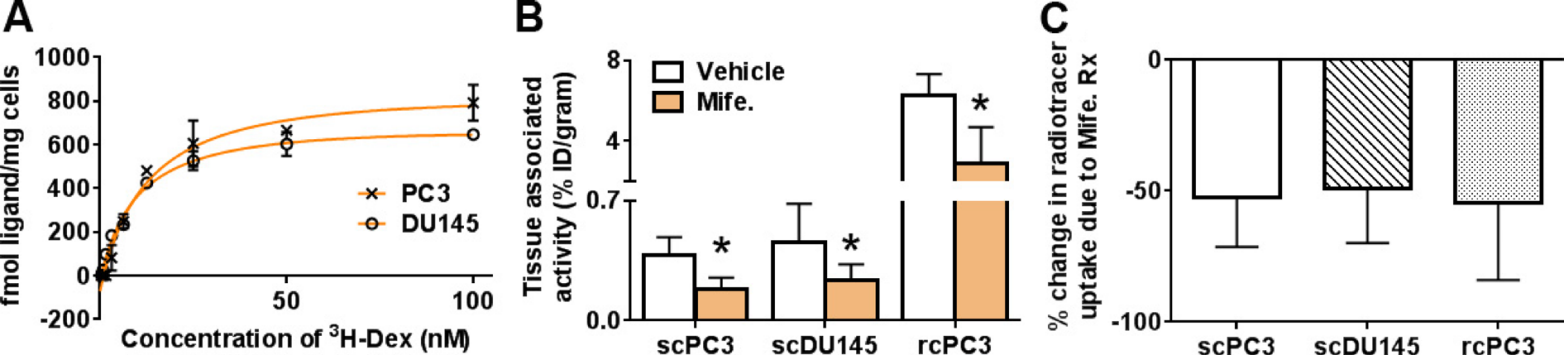
^18^F-GR02 specifically accumulates in subcutaneous and renal capsule prostate cancer tumors (**A**) Saturation binding curves with ^3^H-dexamethasone to determine the GR density per cell reveals that PC3 has a B_max_ value of 1010 fmol/mg, while DU145 has a B_max_ value of 783 fmol/mg, consistent with a previously reported value of 711 fmol/mg [27]. (**B**) Biodistribution data shows that accumulation of ^18^F-GR02 in subcutaneous PC3 and DU145 tumors (scPC3, scDU145) and subrenal capsule PC3 tumors (rcPC3) is suppressed with prior mife. treatment (25 mg/kg). The tumors were implanted in intact male *nu/nu* mice (*n =* 5/treatment arm), and biodistribution data was collected 60 minutes post injection. ^*^P < 0.05 (**C**) Percent changes in radiotracer uptake in mife. treated tumors compared to vehicle shows that mife. suppresses ^18^F-GR02 tumor uptake in all tumor models or microenvironments.

We next tested whether ^18^F-GR02 can detect GR in cancer models. The biodistribution of ^18^F-GR02 (∼11 MBq/mouse) was evaluated in intact male *nu/nu* mice bearing subcutaneous PC3 or DU145 tumors, two prostate cancer models with endogenous and approximately equivalent GR expression (Figure 4A). At one hour post injection, the uptake of ^18^F-GR02 in PC3 and DU145 tumors was 0.37 ± 0.1% ID/g and 0.45 ± 0.2% ID/g, respectively. In both cases, tumor uptake of ^18^F-GR02 was suppressed by four days of prior mife. treatment (25 mg/kg, PC3 = 0.18 ± 0.07% ID/g, *P* = 0.013, DU145 = 0.23 ± 0.09% ID/g, *P* = 0.022, see Figure 4B and Supplementary Figure 8). The biodistribution of ^18^F-GR02 in more highly perfused PC3 tumors implanted in the renal capsule was also evaluated (Supplementary Figures 9 and 10). The uptake of ^18^F-GR02 was ∼16 fold higher in the renal capsule tumor compared to the respective subcutaneous tumor (6.24 ± 1.0% ID/g). Moreover, pretreatment with mife. suppressed radiotracer uptake in the tumor to a statistically significant degree (2.84 ± 1.8% ID/g, *P* = 0.014, Figure 4B). In all cases, the relative suppression of ^18^F-GR02 uptake in tumors due to mife. treatment was equivalent (Figure 4C).

## DISCUSSION

This manuscript outlines the synthesis and preliminary pharmacology of ^18^F-GR02, an experimental radioligand that may provide a more holistic view of GR expression levels *in vivo* with PET. The precursor synthesis was high yielding, the radiochemistry can be automated, and the preparation of ^18^F-GR02 utilizes radiochemistry that is safe for human translation [23]. Small animal PET/CT and biodistribution studies showed that ^18^F-GR02 detected GR rich tissues in intact immunocompetent mice with circulating endogenous corticosteroids. Tissue uptake of the radiotracer was competed by pre-dosing mice with mife., a potent GR antagonist in clinical trials for the treatment of cancer. Radiotracer uptake also increased substantially in many normal tissues within adx mice compared to intact, and radiotracer accumulation in adx mice was blocked with mife. Lastly, ^18^F-GR02 accumulated in a mife.-dependent fashion in PC3 and DU145, two human prostate cancer models with endogenous GR expression (PC3 is also PR null).

In the absence of an imaging tool to detect GR expression levels, the bioactivity of GR agonists or antagonists has been inferred clinically using indirect measures like behavioral changes or alterations in the levels of circulating corticosterone and adrenocorticotropic hormone. The development of ^18^F-GR02 presents the opportunity to study more systematically the pharmacology of GR modulators within specific organs of interest.

While ^18^F-GR02 can detect relative changes in GR protein, it cannot directly measure the transcriptional activity of GR. For castration resistant prostate cancer, GR activity can likely be assessed based on relative changes in serum levels of prostate specific antigen, an AR and GR target gene, or by measuring AR/GR target gene expression changes with PET [24–26]. For other malignancies and human disorders, further research will be required to identify biomarkers reflective of GR activity that can be measured non-invasively.

The uptake of ^18^F-GR02 in normal tissues generally exceeds what was reported for prior GR-targeted radiotracers (summarized in Supplementary Table 2). In some tissues, for example the liver, we observed substantially higher specific binding of ^18^F-GR02 (∼3–10 fold). The specific activity of ^18^F-GR02 is in many cases lower than what was reported previously, suggesting that its improved tissue penetrance is related to its pharmacokinetic properties instead of a higher yielding radiosynthesis. Since the log P of GR02 is considerably lower than other radiofluorinated corticosteroids (1.33, Supplementary Table 1), we are beginning to test the impact of this property on its biodistribution.

Successful detection of subfamily 3 group C nuclear hormone receptors and the pharmacodynamic measurement of receptor occupancy by cognate drugs *in vivo* using 16β- [^18^F]fluoro-5α-dihydrotestosterone [16], 16α- [^18^F]Fluoro-17β-estradiol [17], and 21- [^18^F]Fluoro-16α,17α- [(R)-(1′-α-furylmethylidene) dioxy]-19-norpregn-4-ene-3,20-dione [18] gives confidence that ^18^F-GR02 may also be a clinically useful radiotracer. We are actively performing IND-enabling studies to motivate a first in man study.

## MATERIALS AND METHODS

### Reagents

All chemicals were purchased from Sigma Aldrich and used without further purification. Mifepristone (RU486), Eplerenone, Dihydrotestosterone, 4-OH-Tamoxifen, Dexamethasone, Progesterone were purchased from Sigma Aldrich and reconstituted for cell or animal studies without additional purification. All cell lines were obtained from ATCC and propagated according to manufacturer’s instructions, and verified as mycoplasma negative using the MycoAlert detection kit (Lonza). ^3^H-labeled steroids were purchased from Perkin Elmer and used without further purification.

### Synthesis

All synthetic details and characterization of cold or radiolabeled reaction products can be found in the Supplementary Material.

### Competition binding assays

The K_D_ for subfamily 3 group C nuclear hormone receptors was determined on cells by displacing ^3^H-steroidal agonists for the respective receptor. GR01, GR02, and the “reference” compounds (dexamethasone for GR, dihydrotestosterone for AR, progesterone for PR, 4-OH-tamoxifen for ER, and eplerenone for MR) were added to cells over a concentration range of 10 μM to 1 pM. Each ^3^H-radioligand (dexamethasone for GR, dihydrotestosterone for AR, aldosterone for MR, progesterone for PR, and estradiol for ER) was added at a molar concentration corresponding to 10× the K_D_ of the radioligand for the respective receptor. The K_D_ of GR01 and GR02 was determined using the following cell lines: DU145 (GR), LNCaP-AR (AR), MCF7 (PR, ER), GL261 (mouse GR), and 9L (rat GR).

The cold ligands and ^3^H-steroids were co-incubated on cells in PBS at room temperature for one hour. Following incubation, the cells were washed twice with ice cold PBS and the unbound activity was retained for analysis. The cells were lysed with 1 mL of 1 M NaOH and collected. Bound and unbound fractions were counted in a liquid scintillation counter and expressed as a percentage of the total activity added per equal relative number of cells. To determine non-specific binding, separate treatment arms were established in which excess (>1000 × K_D_) cold reference compound was co-incubated with the experimental ligand and the ^3^H-steroid. These experiments were performed at three concentrations of experimental ligand (1 pM, 100 nM and 10 μM), and a linear extrapolation was used to subtract the non-specific component of binding from each treatment condition. The specific binding component was plotted against the log of the competing ligand and curve fit using a non-linear regression algorithm with PRISM software.

### Saturation binding assay

The number of GR copies per cell was determined in PC3 and DU145 by conducting saturation binding assays with ^3^H-Dexamethasone. 5 × 10^4^ cells were incubated with a range of 9 different concentrations between 0.4 to 100 nM for one hour at room temperature. The non-specific binding was determined at 3 different concentrations (0.4, 12.5 and 100 nM) by co-incubation of separate treatment arms with excess cold dexamethasone (10 μM). Following incubation, the cells were washed twice with ice cold PBS and this fraction was retained for analysis. The cells were lysed with 1 mL of 1M NaOH to collect the bound activity. The soluble and cell associated fractions were counted in a liquid scintillation counter and expressed as a percentage of the total activity added per number of cells. The specific binding was obtained by subtracting the non-specific binding from total. The specific binding was plotted against the concentration of the radioligand using PRISM software. A Rosenthal plot was used to determine the B_max_.

### Log P determination

To a microcentrifuge tube containing 1 mL of octanol:water (1:1 v/v) was added 50 μL of ^18^F-GR02 (total activity = 37 MBq). The solution was vortexed vigorously for 30 sec, and the biphasic layers were allowed to separate. The layers were manually pipetted into new microcentrifuge tubes, and the activity of each fraction was read with a dose calibrator. The water fraction contained 1.7 MBq, and the octanol fraction contained 35.3 MBq.

### Animal experiments

All animal experiments were conducted with prior approval from IACUC at UCSF. Five to seven week old male *nu/nu*, intact or adx C57BL6/J mice were purchased from Charles River. Adx mice were provided drinking water supplemented with 0.9% NaCl (aq.). Male *nu/nu* mice received a subcutaneous bolus of 1 × 10^7^ PC3 or DU145 cells in Matrigel and growth media (1:1 *v/v*) in the flank for tumor imaging studies with ^18^F-GR02. For subrenal capsule tumor implants, 4–6 week old male Rag2 knockout mice (Taconic Farms) were placed under anesthesia with ∼2% isoflurane. A half centimeter dorsal midline incision was performed, and one kidney was gently pulled though the incision by applying pressure to the muscle wall. The kidney capsule was lifted from the parenchyma of the kidney, and PC3 cells (5 × 10^6^ in 50 μL PBS) were injected into the pocket under the capsule. The kidney was placed back and the skin incision was closed using 3 surgical sutures. The mice were treated once with Carprofen (5–10 mg/kg) to ease recovery. Mice were observed over 24 hours for signs of post-operative pain, bleeding and/or other complications. Tumor progression was followed for 7–14 days after surgery with a 14 T Agilent small animal MRI to identify tumors of suitable size to conduct the biodistribution study.

### Statistics

All statistical analysis was performed used PRISM software. Statistically significant differences in the data were determined using an unpaired Student’s *t* test. Changes at the 95% confidence level (*P* < 0.05) were qualified as statistically significant.

### Small animal PET/CT

All radiotracers were administered via tail vein injection in < 300 μL PBS. Intact male C57BL6/J or *nu/nu* mice were treated with vehicle or mife. (25 mg/kg) via oral gavage for four days prior to injection with ∼11 MBq of ^18^F-GR02. Mice were anesthetized and imaged with a Siemens Inveon small animal PET/CT at dedicated time points post injection. Each mouse was imaged until 20 million coincident events were collected. All imaging data was reconstructed, decay corrected, and analyzed with AMIDE or ASIPro software. Maximum intensity projections were generated with ASIPro software. ASIPro software was also used to draw manual two dimensional regions of interest to calculate SUV data for static or dynamic acquisitions.

### Biodistribution studies

At dedicated time points post injection, mice were humanely euthanized with CO_2_ (g) asphyxiation and blood and tissues were removed, washed, dried and weighed. The activity associated with each tissue was measured with a gamma counter. The data was decay corrected and expressed as a percentage of the injected dose per gram of tissue with PRISM software.

## SUPPLEMENTARY MATERIALS FIGURES AND TABLES



## Abbreviations

GR: glucorticoid receptor
AR: androgen receptor
ER: estrogen receptor
PR: progesterone receptor
MR: mineralocorticoid receptor
mife: mifepristone
I: intact
Adx: adrenalectomized
Lg. Int.: large intestine
Sm. Int.: small intestine
KOH: potassium hydroxide
TBS: tert-butyl silane
DMF: N; N-dimethyl-formamide
TBAF: tetra-butyl ammonium fluoride
THF: tetrahydrofuran
MsCl: methanesulfonyl chloride
TEA: triethylammonia
DCM: dichloromethane
pyr: pyridine
NaN_3_: sodium azide
NaI: sodium iodide
MeOH: methanol
NMP: N-methyl-2-pyrrolidine
DIPEA: N;N-diisopropylethylamine
