# Supplementary material for: Measuring glucocorticoid receptor expression in vivo with PET
Source: Oncotarget. 2018 Apr 17;9(29):20399–408. doi: 10.18632/oncotarget.24911 (PMC5945515; doi:10.18632/oncotarget.24911)
Supplement: Supplementary file 1 [file oncotarget-09-20399-s001.pdf]

Measuring glucocorticoid receptor expression *in vivo* with PET

SUPPLEMENTARY MATERIALS

General synthetic methods

All chemicals, including anhydrous solvents, were purchased from Sigma Aldrich and used without further purification, unless otherwise noted. Deuterated solvents for NMR spectroscopy were purchased from Cambridge Isotopes Laboratories. NMR spectra were recorded on a Bruker Advance III 400 MHz spectrometer at the UCSF NMR lab, and spectra were analyzed using Mestrelab software. High resolution mass spectra were recorded on a Bruker microTOF II at the University of Notre Dame's Mass Spectrometry and Proteomics core facility. Fluorine-18 was obtained as a NaF salt from the cyclotron core at UCSF.

Synthetic methods for GR01 and GR02

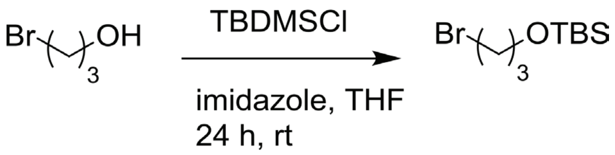

To a solution of 3-bromopropanol (10 mL, 114 mmol, 1 eq.) in anhydrous tetrahydrofuran (100 mL) was added tert-butyl-dimethylsilyl chloride (22.2 g, 149 mmol, 1.3 eq.) followed by imidazole (23.2 g, 343 mmol, 3 eq.)

The reaction was stirred at room temperature for 24 hours under argon (g). The reaction was concentrated under reduced pressure and resuspended in water (100 mL) and extracted with dichloromethane (3 × 20 mL). The organic portions were combined, washed with brine (60 mL), dried over Na_2SO_4, filtered, and concentrated under reduced pressure to afford 3-bromopropyl-*tert*-butyldimethylsilyl ether as a clear and colorless oil (28 g, 110 mmol, 97% yield). ^{1}H NMR ($CDCl_3$): δ 0.06 (s, 6H); 0.89 (s, 9H); 1.99-2.07 (m, 2H); 3.51 (t, 2H, J = 3.2 Hz); 3.73 (t, 2H, J = 2.8 Hz).

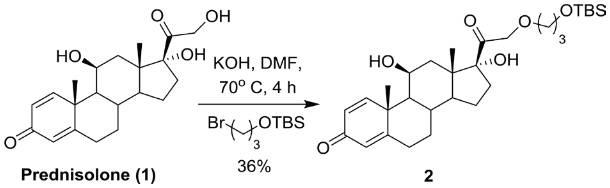

To a solution of prednisolone (1.5 g, 4.16 mmol, 1 eq.) in a 1:1 mixture of 3-bromopropyl-*tert*-butyldimethylsilyl ether (4 mL, 10 eq) and anhydrous dimethyl formamide (4 mL) was added freshly pulverized KOH (700 mg, 12.5 mmol, 3 eq.). The reaction was stirred at 70° C for 4 hours under argon (g). The reaction was diluted with ethyl acetate (80 mL) and washed with saturated NH_4Cl (aq) solution (5 × 20 mL), washed with brine (1 × 20 mL), dried over Na_2SO_4, filtered, and concentrated under reduced pressure. The residue was purified by silica chromatography (hexanes:ethyl acetate) to afford 2 as a white solid (798 mg, 1.5 mmol, 36% yield). ^{1}H NMR (DMSO-D6) δ 0.1 (6H, s) 0.9 (3H, s); 1 (9H, s); 1.0 (1H, m); 1.3 (1H, m); 1.4 (3H, s); 1.4 (1H, m); 1.6 (3H, m); 1.9 (1H, m); 2.0 (4H, m) 2.3 (1H, m); 2.6 (6H, m); 3.6 (1H, t, J = 6.2 Hz); 3.7 (1H, t, J = 6.2 Hz); 4.4 (1H, bs); 5.9 (1H, bs); 6.2 (1H; d, J = 11.3 Hz); 7.3 (1H, d, J = 9.8 Hz). HR-MS: Calculated for $C_{30}H_{48}O_6Si$ 533.329 $(M+H)^+$ determined 533.331.

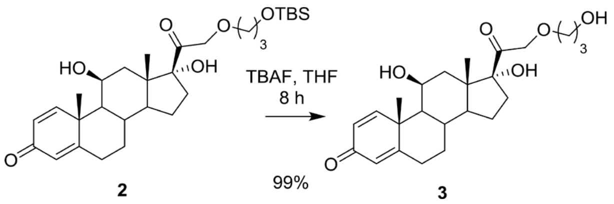

To a solution of 2 (750 mg, 1.4 mmol, 1 eq.) in tetrahydrofuran (14 mL) was added 1M tetra-butyl ammonium fluoride solution (1.8 mL, 1.8 mmol, 1.3 eq.). The reaction was stirred at room temperature for 8 hours. The reaction with concentrated under reduced pressure and passed through a silica plug to afford **3** as a white solid (580 mg, 1.39 mmol, 99% yield). ^{1}H NMR (DMSO-D6) δ 1.0 (3H, s); 1.1 (1H, m); 1.3 (1H, m); 1.4 (3H, s); 1.4 (1H, m); 1.6 (3H, m); 1.9 (1H, m); 2.0 (4H, m) 2.3 (1H, m); 2.6 (6H, m); 3.7 (2H, dd, J = 6.6, 7.3 Hz); 4.4 (1H, m); 5.9 (1H, bs); 6.2 (1H; d, J = 10.5 Hz); 7.3 (1H, d, J = 9.2 Hz). HR-MS: Calculated for $C_{24}H_{34}O_6$ 419.242 $(M+H)^+$ determined 419.242.

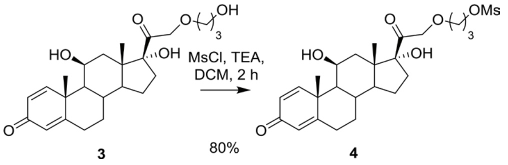

To a solution of 3 (42 mg, 100 µmol, 1 eq.) in anhydrous dichloromethane (1 mL) was added methanesulfonyl chloride (15.5 µL, 200 µmoles, 2 eq.) followed by triethylamine (42 µL, 300 µmoles, 3 eq.). The reaction was stirred at room temperature for 2 hours. The reaction was diluted with ethyl acetate (10 mL) and washed with 1M HCl (aq.) solution (3 × 2 mL), washed with brine (1 × 2 mL), dried over Na_2SO_4, filtered, concentrated to afford 4 as a white solid (40 mg, 80 µmoles, 80% yield). Compound 4 was used without any further purification.

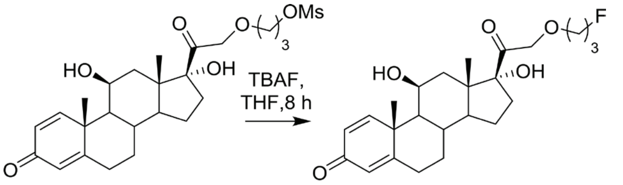

4 72% **GR01 (5)**

To a solution of 4 (40 mg, 80 µmol, 1 eq.) in anhydrous tetrahydrofuran (1.6 mL) was added 1M tetrabutyl ammonium fluoride solution (104 µL, 104 µmol, 1.3 eq.). The reaction was stirred at room temperature for 8 hours. The reaction with concentrated under reduced pressure and purified by silica chromatography (hexanes:ethyl acetate) to afford 5 as a white solid (24 mg, 58 µmol, 72% yield). ^{1}H NMR (DMSO-D6) δ 0.8 (3H, s); 0.9 (1H, m); 1.0 (1H, m); 1.3 (1H, m); 1.4 (3H, s); 1.4 (1H, m); 1.6 (3H, m); 1.9 (1H, m); 2.0 (4H, m) 2.3 (1H, m); 2.6 (6H, m); 3.9 (2H, m); 4.5 (1H, m); 5.9 (1H, bs); 6.2 (1H; d, J = 9.9 Hz); 7.3 (1H, d, J = 9.3 Hz). HR-MS: Calculated for $C_{24}H_{33}FO_5$ 421.238 $(M+H)^+$ determined 421.238.

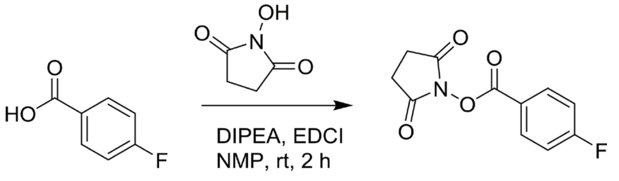

89%

To a solution of fluorobenzoic acid (1.4 g, 10 mmol, 1 eq.) and N-hydroxysuccinimide (1.27 g, 11 mmol, 1.1 eq.) in anhydrous N-methyl-2-pyrrolidone (30 mL) was added *N*-(3-dimethylaminopropyl)-*N'*-ethylcarbodiimide hydrochloride (2.3 g, 12 mmol, 1.2 eq.) followed by *N,N*-diisopropylethylamine (3.5 mL, 20 mmol, 2 eq.). The reaction was stirred at room temperature for 2 hours. The reaction was diluted with ethyl acetate (300 mL) and washed with 1M HCl (aq) solution (3 × 60 mL), washed with brine (1 × 60), dried over Na_2SO_4, filtered, concentrated to afford *N*-succinimidyl-4-fluorobenzoate as a white solid (2.1 g, 8.9 mmol, 89% yield). ^{1}H NMR (400 MHz, $CDCl_3$) δ (ppm) 2.9 (s, 4H); 7.2 (m, 2H); 8.1 (m, 2H). The identity of the product was verified by comparing the ^{1}H NMR data to the spectrum previously reported in the literature [1].

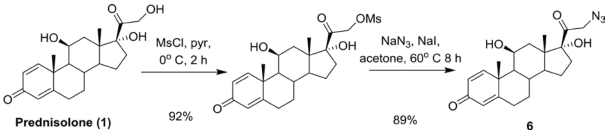

Prednisolone (1) 92% 89% **6**

The C21 primary alcohol on prednisolone was converted to a methanesulfonyl ester following a previously described protocol[2]. To a solution of prednisolone (1.5 g, 4.16 mmol, 1 eq.) in pyridine (10 mL) at 0° C was added methanesulfonyl chloride (1.5 mL, 19.4 mmol, 4.7 eq.). The reaction was stirred for 2 hours at 0° C. The reaction was added dropwise to a rapidly stirring solution of ice water (100 mL) and stirring was continued until the ice completely melted. The solution was filtered under reduced pressure and the collected precipitate was washed with cold water and dried overnight under high vacuum.

The white solid was suspended in methyl acetate (40 mL) and NaI (2.5 g, 16.6 mmol, 4 eq.) was added followed by NaN_3 (1.35 g, 20.8 mmol, 5 eq.). The reaction was stirred at 60° C overnight. The reaction was added dropwise to a rapidly stirring solution of ice water (400 mL) and stirring was continued until the ice completely melted. The solution was filtered under reduced pressure and the collected precipitate was washed with cold water and dried overnight under high vacuum to afford 6 as a tan solid (1.43 g, 3.7 mmol, 89% yield). ^{1}H NMR (DMSO-D6) δ 0.8 (3H, s); 0.9 (1H, m); 1.0 (1H, m); 1.3 (1H, m); 1.4 (3H, s); 1.5 (1H, m); 1.7 (3H, m); 1.9 (1H, m); 2.0 (2H, m) 2.3 (1H, m); 2.6 (2H, m); 3.9 (1H, d, J = 18.8 Hz); 4.2 (1H, s); 4.5 (1H, d, J = 18.8 Hz); 5.9 (1H, s); 6.2 (1H; d, J = 10.2 Hz); 7.3 (1H, d, J = 10.9 Hz). HR-MS: Calculated for $C_{21}H_{27}N_3O_4$ 386.207 $(M+H)^+$ determined 386.208.

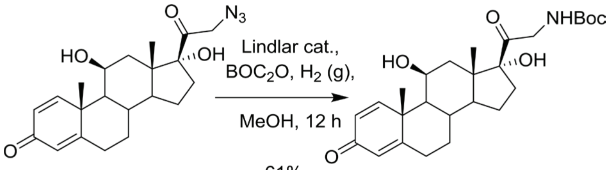

6 61% **7**

To a solution of **6** (1 g, 2.6 mmol, 1 eq.) and di-tertbutyl-dicarbonate (1.1 g, 5.2 mmol, 2 eq.) in anhydrous methanol (13 mL) was added Lindlar catalyst (200 mg, 20% by wt.). The reaction was evacuated and sparged with H_2 (g) thrice and stirred at room temperature for 12 hours under 1 atm of H_2 (g). The reaction was concentrated onto Celite™ and purified by silica chromatography (hexanes:ethyl acetate) to afford 7 as a white solid (735 mg, 1.6 mmol, 61% yield). ^{1}H NMR (DMSO-D6) δ 0.8 (3H, s); 0.9 (1H, m); 1.0 (1H, m); 1.3 (1H, m); 1.4 (3H, s); 1.4 (1H, m); 1.5 (9H, s); 1.6 (3H, m); 1.9 (1H, m); 2.0 (2H, m) 2.3 (1H, m); 2.6 (2H, m); 3.9 (1H, d, J = 19.2 Hz); 4.2 (1H, m); 4.5 (1H, d, J = 19.2 Hz); 5.9 (1H, s); 6.2 (1H; d, J = 10.3 Hz); 7.3 (1H, d, J = 10.0 Hz). HR-MS: Calculated for $C_{26}H_{37}NO_6$ 460.269 $(M+H)^+$ determined 460.270.

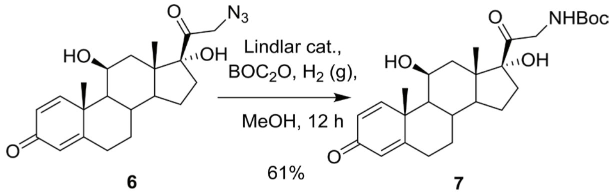

To a solution of 7 (700 mg, 1.5 mmol) in anhydrous dichloromethane (7.5 mL) was added trifluoroacetic acid (7.5 mL). The reaction was stirred for 3 hours at room temperature. The reaction was concentrated under reduced pressure to afford 8 as a white solid (710 mg, 1.5 mmol, 99%). ^1H NMR (DMSO-D6) δ 0.8 (3H, s); 0.9 (1H, m); 1.0 (1H, m); 1.3 (1H, m); 1.4 (3H, s); 1.4 (1H, m); 1.6 (3H, m); 1.9 (1H, m); 2.0 (2H, m) 2.3 (1H, m); 2.6 (2H, m); 4.0 (1H, dd, J = 4.5, 20.2 Hz); 4.4 (1H, dd, J = 4.5, 20.2 Hz); 4.5 (1H, bs); 5.9 (1H, bs); 6.2 (1H; d, J = 10.0 Hz); 7.3 (1H, d, J = 10.7 Hz). HR-MS: Calculated for $C_{21}H_{30}NO_4^+$ 360.217 $(M+H)^+$ determined 360.218.

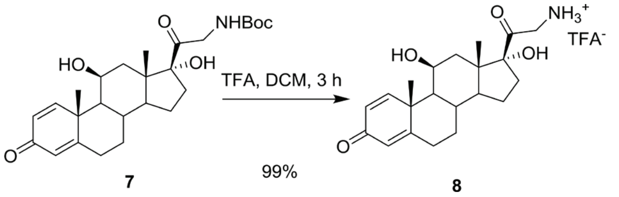

To a solution of 8 (47 mg, 100 μmol, 1 eq.) in N-methyl-pyrrolidone (1 mL) was added N-succinimidyl-4-fluorobenzoate (26 mg, 110 μmol, 1.1 eq.) followed by *N*,*N*-diisopropylethylamine (35 μL, 200 μmol, 2 eq.). The reaction was stirred at room temperature for 8 hours. The reaction was diluted with ethyl acetate (10 mL) and washed with 1M HCl (aq) solution (3 × 2 mL), washed with brine (1 × 2 mL), dried over Na_2SO_4, filtered, and concentrated under reduced pressure. The residue was purified by silica chromatography (Hexanes:ethyl acetate) to afford 9 as a white solid (40 mg, 83 μmol, 83%). ^1H NMR (DMSO-D6) δ 0.8 (3H, s); 0.9 (1H, m); 1.0 (1H, m); 1.3 (1H, m); 1.4 (3H, s); 1.4 (1H, m); 1.6 (3H, m); 1.9 (1H, m); 2.0 (2H, m) 2.3 (1H, m); 2.6 (2H, m); 4.1 (1H, d, J = 19.5 Hz); 4.3 (1H, m); 4.5 (1H, d, J = 19.5 Hz); 5.9 (1H, bs); 6.1 (1H; dd, J = 1.9, 10.2 Hz); 7.2 (2H, m); 7.3 (1H, d, J = 10.2 Hz); 8.2 (2H, m). HR-MS: Calculated for $C_{28}H_{32}FNO_5$ 482.233 $(M+H)^+$ determined 482.235.

Radiosynthesis of ^{18}F-GR01

[^{18}F]-fluoride anion (3.7–18.5 GBq) was eluted off a QMA Sep-Pak cartridge using 0.5 mL of a $K_{2.2.2}/KHCO_3$ solution (11 mg/mL of $K_{2.2.2}$, 1 mg/mL of K_2CO_3, 9:1 *v:v* acetonitrile:H_2O). The resulting solution was subjected to 3 drying cycles at 115°C under a gentle stream of nitrogen. Compound 4 (~5 mg) was dissolved in anhydrous DMSO (800 μL) and added to [^{18}F]-fluoride, and the resulting solution was sealed and heated at 130° C for 20 min. ^{18}F-GR01 was isolated via semi-preparative reverse phase-HPLC coupled to a radiation detector (5%-95% gradient of acetonitrile in H_2O with 0.1% formic acid.) The product fraction isolated off of the HPLC was diluted to ~30 mL in H_2O and loaded onto a C18-light Sep-Pak cartridge. The product fraction isolated off of the HPLC was diluted to ~30 mL in H_2O and loaded onto a C18-light Sep-Pak cartridge preconditioned with ethanol (5 mL) and H_2O (5 mL). The cartridge was washed with 10 mL H_2O and the activity was eluted with ethanol (500 μL). The resulting solution was then concentrated for 1 h at 50° C under reduced pressure, and ^{18}F-GR02 was then dissolved in a solution of 15% ethanol (aq.) for animal studies. The radiotracer was confirmed to be >99% radiochemical purity prior to administration in animals.

Radiosythesis of ^{18}F-GR02

[^{18}F]-fluoride anion (3.7–18.5 GBq) was eluted off a QMA Sep-Pak cartridge using 0.5 mL of a $K_{2.2.2}$/ $KHCO_3$ solution (11 mg/mL of $K_{2.2.2}$, 1 mg/mL of K_2CO_3, 9:1 *v:v* acetonitrile:H_2O). The resulting solution was subjected to 3 drying cycles at 115° C under a gentle stream of nitrogen. Ethyl 4-(tri-methylammonium triflate) benzoate (~5 mg) was dissolved in anhydrous acetonitrile (800 μL) and added to [^{18}F]-fluoride, and the resulting solution was sealed and heated at 90° C for 10 min. A 25% solution of tetra-propylammonium hydroxide (20 μL) in 0.5 mL acetonitrile was added in the reaction vial, and the solution was sealed and heated at 90° C for 8 minutes. The solution was dried down and washed with acetonitrile three times. A solution of *N*,*N*,*N′*,*N′*-tetra-methyl-O-(*N*-succinimidyl) uronium tetrafluoroborate (30 mg) in acetonitrile (1 mL) was added to the reaction vial and heated for 8 minutes at 90° C. *N*-succinimidyl 4-[^{18}F]-fluorobenzoate was then eluted off a HLB plus Sep-Pak cartridge in acetonitrile.

N-succinimidyl 4-[^{18}F]-fluorobenzoate was reacted with 8 (2.5 mg) in a solution of 1:1 dimethyl sulfoxide:acetonitrile (500 μL total volume) with triethylamine (25 μL) at 90° C for 10 min. ^{18}F-GR02 was isolated via semi-preparative reverse phase-HPLC coupled to a radiation detector (20–60% gradient of acetonitrile in H_2O with 0.1% formic acid). The product fraction isolated off of the HPLC was diluted to ~30 mL in H_2O and loaded onto a C18-light Sep-Pak cartridge. The Sep-Pak cartridge was washed with ethanol (5 mL) and H_2O (5 mL), and then the collected activity was added onto the Sep-Pak cartridge and was washed with 10 mL H_2O and the activity was eluted with ethanol (0.5 μL). The resulting solution was then concentrated for 1 h at 50° C under reduced pressure, and ^{18}F-GR02 was then dissolved in a solution of 15% ethanol (aq.) for animal studies. The radiotracer was >99% pure prior to administration in animals.t

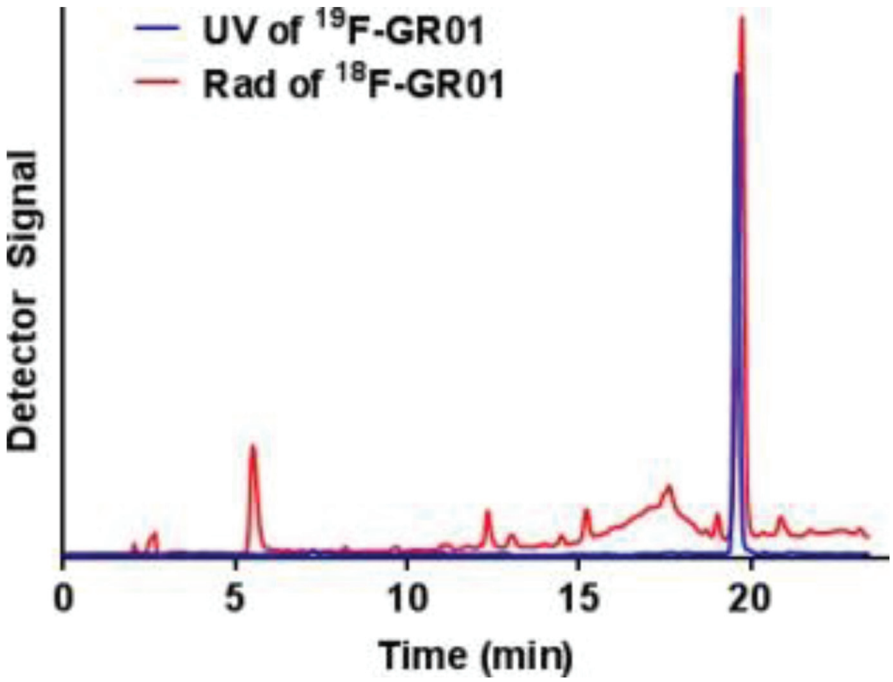

Supplementary Figure 1: Coupled radioactivity/UV-vis reverse phase HPLC trace for the crude reaction that conferred ^{18}F-GR01. The HPLC mobile phase was 5–95% gradient of acetonitrile in H_2O with 0.1% formic acid. The peak at 19 min corresponds to GR01.

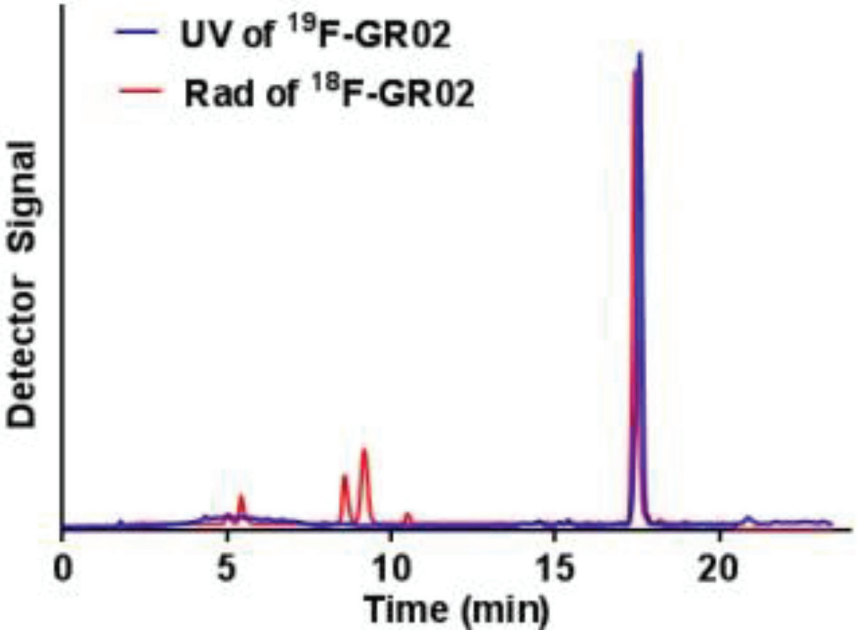

Supplementary Figure 2: Coupled radioactivity/UV-vis reverse phase HPLC trace for the crude reaction that conferred ^{18}F-GR02. The HPLC mobile phase was a 20–60% gradient of acetonitrile in H_2O with 0.1% formic acid. The peak at 17 min corresponds to GR02.

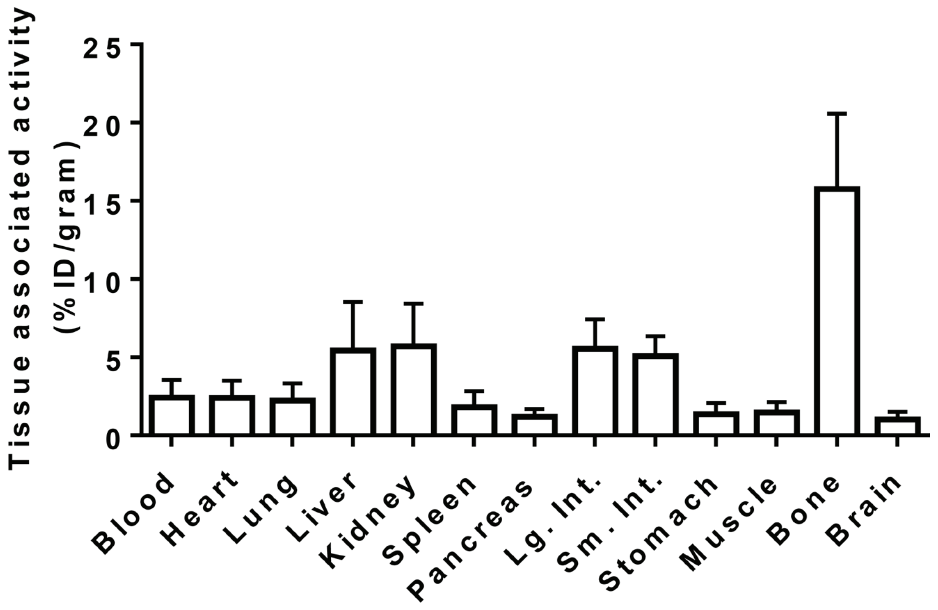

Supplementary Figure 3: Biodistribution data collected for ^{18}F-GR01 in tumor naïve intact male C57BL6/J mice at one hour post injection, ~11 MBq/mouse. The data represents the mean and standard deviation of *n* = 5 mice per treatment arm.

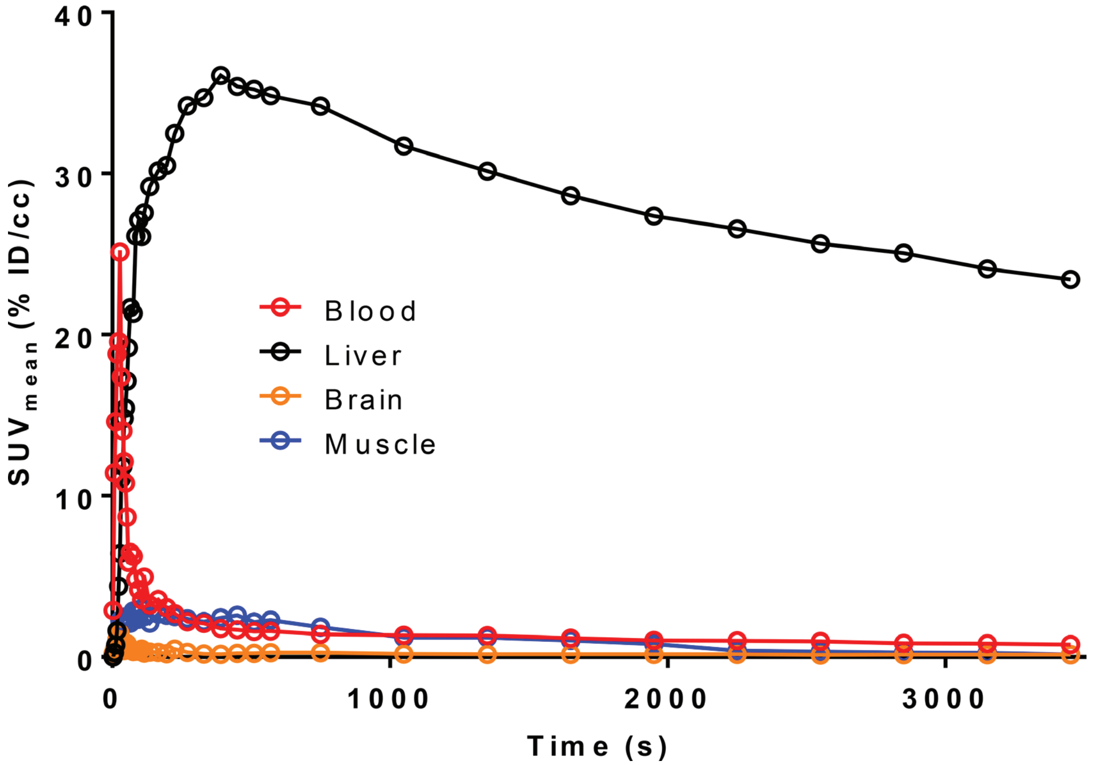

Supplementary Figure 4: A time activity curve derived from a dynamic small animal PET scan collected in one tumor naïve intact male C57BL6/J mouse injected with ~11 MBq of ^{18}F-GR02. Two dimensional regions of interest were drawn manually over the respective tissue or compartment using ASIPro software. The SUV data were plotted using PRISM.

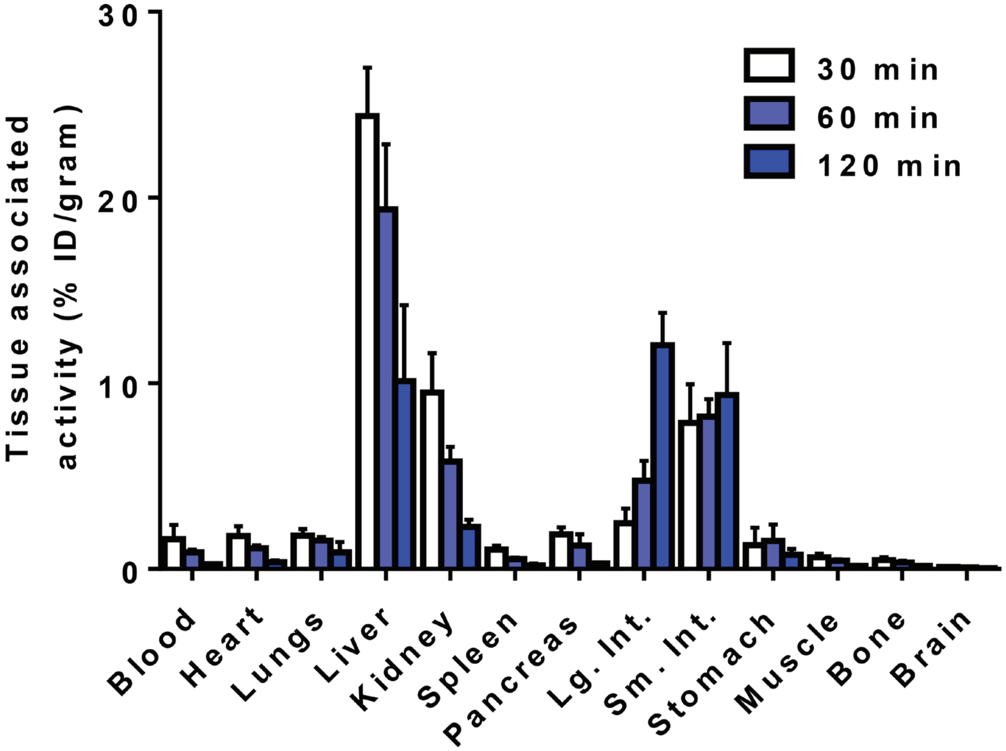

Supplementary Figure 5: Biodistribution data for ^{18}F-GR02 (~11 MBq/mouse) showing a more complete repertoire of tissues from tumor naïve intact male C57BL6/J mice over time. The data represents the mean and standard deviation of 4 replicates per time point.

Vehicle	Mean	Std Dev	Size (mm^3)
	8.99	0.7	9.44
	6.43	0.5	4.71
	7.77	0.4	3.79
	7.73	0.8	5.88
**Mife.**	17.11	1.1	10.50
	13.22	1.0	8.52
	13.64	0.6	13.94
	15.73	2.0	10.30

Supplementary Figure 6: A chart showing the SUV$_{mean}$, standard deviation and the size of the region of interest used to quantify liver uptake of ^{18}F-GR02 in intact male C57BL6/J mice treated with vehicle or mifepristone. The SUV data were calculated using Amide software and decay corrected images. "Size" refers to the dimensions of the manually drawn circular region of interest.

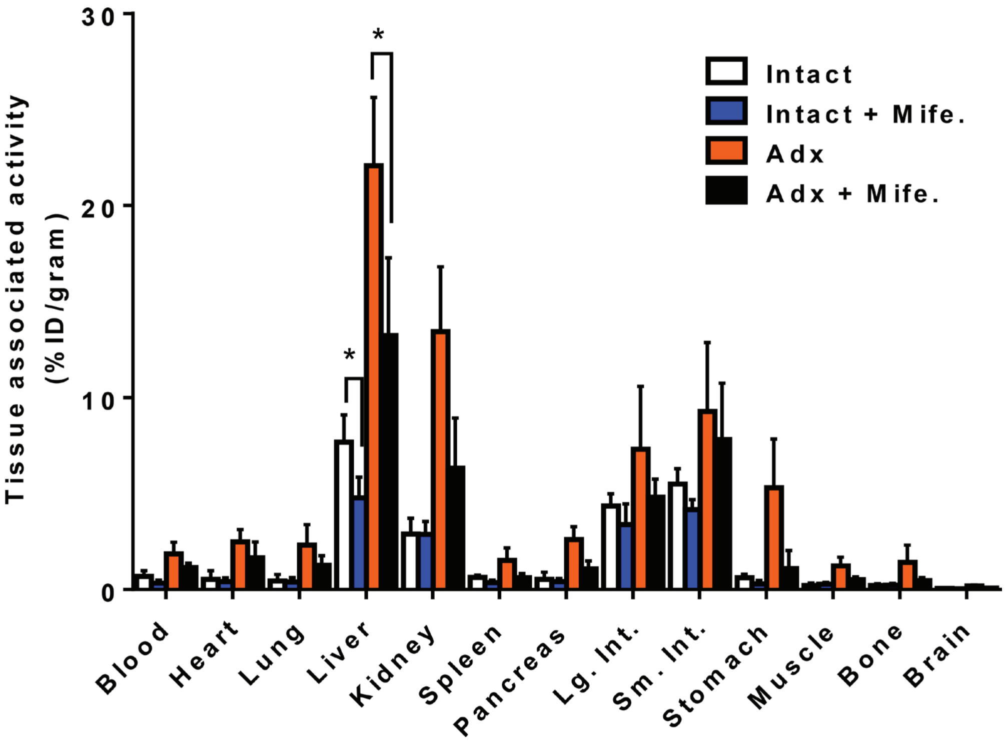

Supplementary Figure 7: Biodistribution data collected for ^{18}F-GR02 in tumor naïve intact C57BL6/J mice treated with vehicle, intact mice treated with mifepristone for four days prior to ^{18}F-GR02, and adx mice treated with vehicle or mifepristone four days prior to ^{18}F-GR02. All data were collected at one hour post injection. The data represent the mean and standard deviation of $n = 5$ mice per treatment arm. $^{*}P < 0.01$

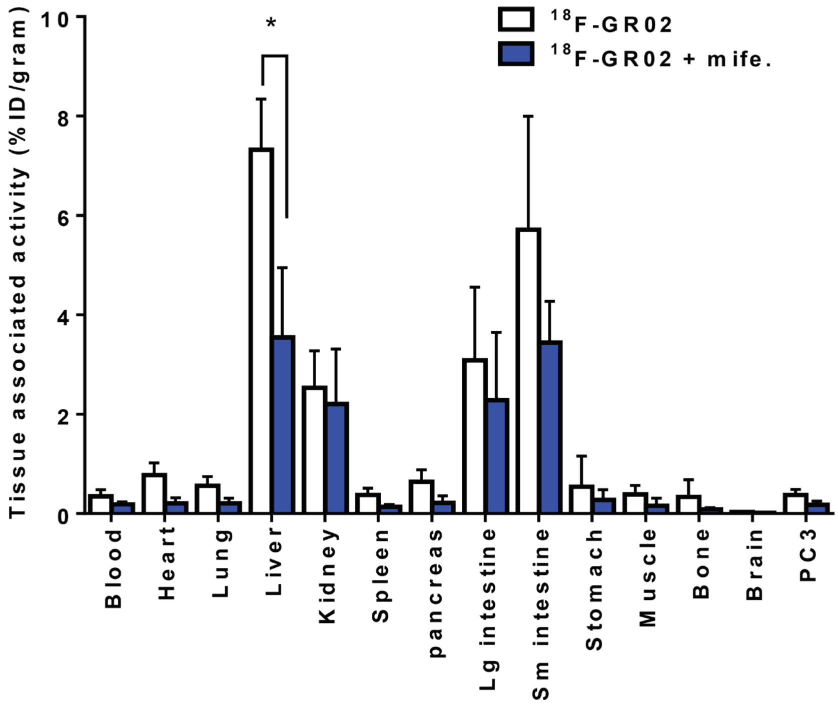

Supplementary Figure 8: Biodistribution data 60 minutes post injection in male nu/nu mice bearing subcutaneous PC3 xenografts treated with vehicle or mifepristone via gavage for 4 days prior to injection with ^{18}F-GR02. The data represent the mean and standard deviation of $n = 5$ mice per treatment arm. The biodistribution in normal tissues is representative of what was observed in nu/nu mouse cohorts bearing subcutaneous DU145 or subrenal capsule PC3 tumors. $^{*}P < 0.01$

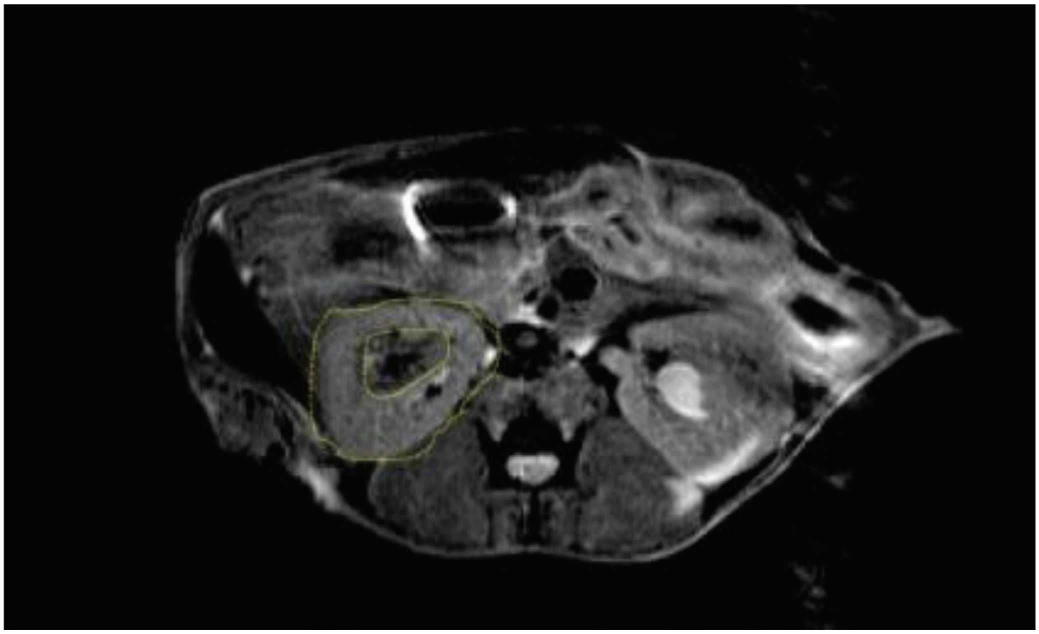

Supplementary Figure 9: A T2 weighted proton axial image (TR/TE = 1200/20 ms) obtained on a microimaging 14 T Agilent spectrometer with a 32 × 32 mm field of view and 256 × 256 matrix points. The yellow dotted lines encapsulate the tumor that has completely infiltrated the kidney excluding the renal pelvis.

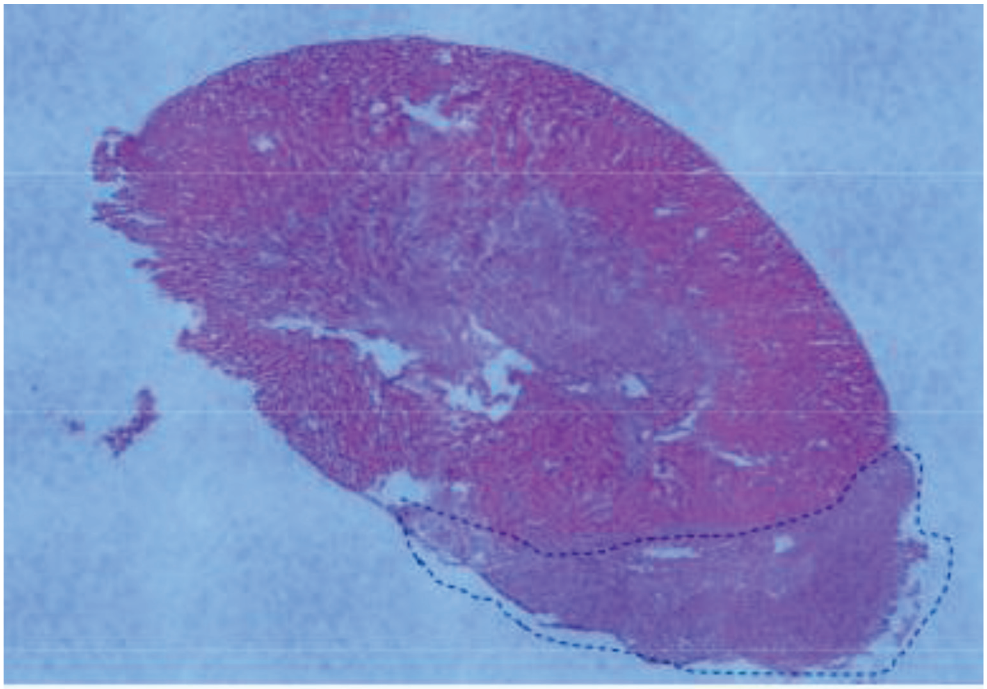

Supplementary Figure 10: H&E staining shows a cross section of a representative tumor-bearing mouse kidney. The dashed black line outlines the tumor. The image is represented at 4× magnification.

Supplementary Table 1: A summary of the properties of the radiochemical syntheses and the log *P* values for all GR radioligands that have been evaluated in small animal models

Compound	Rxn Time (min)	RCY	SA (Ci/ µmol)	K_D (nM)	$LogP_{o/w}$	PMID
21-[^{18}F]-prednisone	70–80	2–8%	2.5	NR	2.1	3495649
3'-[^{18}F]-RU28362	NR	14–27%	2.45	6.4	NR	1526811
21-[^{18}F]-deoxytriamcinolone acetonide	NR	0.60%	0.04	2.1	NR	1526811
[^{18}F]-fluoro-ORG6141	NR	10%	0.2–1	NR	2.66	8547889
[^{18}F]-RU52461	140	12–30%	0.9–1.5	NR	NR	1601670
[^{18}F]-2	80	3–4%	0.8–1.2	34	3.87	15713375
[^{11}C]-Al-438	35	30%	0.27–0.4	8.1	6.13	17499506
[^{18}F]-GR01	50–70	2%	ND	3.8	ND	N/A
[^{18}F]-GR02	50–70	20–30%	0.9–1	15.9	1.33	N/A

Abbreviations: Rxn = reaction, RCY = radiochemical yield, SA = specific activity, NR = not reported, ND = not determined, N/A = not applicable.

Supplementary Table 2: A summary of prior biodistribution data in the literature, including ^{18}F-GR02 data from this study

Compound	Host	Blood	Heart	Lung	Liver	Kidney	Spleen	Stomach	Muscle	Bone	Brain
21-[^{18}F]-pred.	Rt (I)		0.2	0.25	1.7	0.6	0.2	0.2	0.15		0.05
3'-[^{18}F]-RU28362	Rt (A)	0.38 ± 0.02			2.48 ± 0.4	1.23 ± 0.1				0.80 ± 0.04	0.17 ± 0.03
21-[^{18}F]-DA	Rt (A)	0.19 ± 0.05								1.43 ± 0.2	1.14 ± 0.1
[^{18}F]-ORG6141	Rt (A)	0.75	0.5	0.9	1	1	0.25	0.25	0.15	1	
[^{18}F]-RU52461	Rt (I)	0.22 ± 0.02	0.25 ± 0.03	0.34 ± 0.04	1.13 ± 0.06	0.61 ± 0.02			0.17 ± 0.02		0.38 ± 0.02
[^{18}F]-2	Rt (I)	0.06 ± 0.00		0.60 ± 0.1	1.44 ± 0.4	0.66 ± 0.1	0.41 ± 0.06		0.32 ± 0.05	0.33 ± 0.07	0.24 ± 0.04
[^{11}C]-Al-438	Rt (I)	0.60 ± 0.2	0.75 ± 0.15	1.11 ± 0.2	2.54 ± 0.5	1.19 ± 0.3	1.06 ± 0.2		0.59 ± 0.08	0.88 ± 0.2	2.30 ± 0.2
[^{18}F]-GR02	Ms (I)	0.83 ± 0.3	0.81 ± 0.5	0.64 ± 0.4	7.98 ± 1.5	3.96 ± 1.9	0.63 ± 0.1	0.62 ± 0.1	0.36 ± 0.3	0.28 ± 0.1	0.06 ± 0.02
[^{18}F]-GR02	Ms (A)	2.36 ± 1.6	2.39 ± 0.6	2.15 ± 1.0	21.83 ± 3.3	13.42 ± 3.1	1.41 ± 0.6	5.54 ± 2.4	1.15 ± 0.4	1.25 ± 0.8	0.17 ± 0.05

Values shaded in grey indicate tissue associated activity that could be suppressed with a blocking treatment to a statistically significant extent ($P < 0.05$). Empty entries represent data that was not collected and reported. In all cases except for RU28362, data was collected 60 minutes post injection. Biodistribution data for RU28362 was collected 30 minutes post injection. PMID references for the data are listed in Supplementary Table 1. Abbreviations: Rt = rat, Ms = mouse, I = intact, A = adrenalectomized, pred. = prednisone, DA = deoxytriamcinolone acetonide, N/A = not applicable.

REFERENCES

1. Vaidyanathan G, Zalutsky MR. Labeling proteins with fluorine-18 using N-succinimidyl 4-[18F]fluorobenzoate. International journal of radiation applications and instrumentation Part B, Nucl Med Biol. 1992; 19:275–281.
2. Ma K, Hu M, Qi Y, Qiu L, Jin Y, Yu J, Li B. Structure-transfection activity relationships with glucocorticoid-polyethyl-enimine conjugate nuclear gene delivery systems. Biomaterials. 2009; 30:3780–3789.
